# Characterization of the complete mitochondrial genome of a harvesting ant *Messor structor* (Hymenoptera: Formicidae: Myrmicinae)

**DOI:** 10.1080/23802359.2022.2079105

**Published:** 2022-06-02

**Authors:** Xin-Min Zhang, Xiu Han, Xia Liu, Zheng-Hui Xu

**Affiliations:** Key Laboratory of Forest Disaster Warning and Control in Yunnan Province, College of Biodiversity Conservation, Southwest Forestry University, Kunming, Yunnan, China

**Keywords:** *Messor structor*, complete mitochondrial genome, phylogeny, Formicidae

## Abstract

*Messor structor* (Latreille, 1798) is a keystone ant species in the genus *Messor* (Formicidae: Myrmicinae). Here, we reported the complete mitochondrial genome of *M*. *structor*. The circular mitogenome of *M*. *structor* is 17628 bp including 13 protein-coding genes, two ribosomal RNA genes, 22 transfer RNA genes, and a control region. The base composition was AT-biased (84.07%). Phylogenetic analysis suggests that it is closely related to *Aphaenogaster famelica*. The mitochondrial genome of *M*. *structor* will be a good source for understanding molecular evolutionary studies of this species and related ant species.

The genus *Messor* Forel, 1890 is a moderately large genus, more than 126 species in the worldwide were recognized, which are mainly distributed in the Palearctic, Afrotropical, and Oriental regions (Salata and Borowiec 2019; Bolton 2021). *Messor* species are granivorous and play an important role in ecosystem maintenance, and plant seeds dispersal. Most of species are reported from open and arid habitats, such as semideserts, and deserts, grasslands, savannahs, and phryganas (Bolton 1982; Salata and Borowiec 2019). A series of taxonomic revisions of the genus *Messor* have been occurred (Santschi 1917, 1923, 1927; Kuznetsov-Ugamsky 1927; Finzi 1929; Steiner et al. 2018; Salata and Borowiec 2019). Since current partial revisions were only confined to some species or to some geographic regions (Arnol’di 1977; Bolton 1982), thus the present taxonomy of this genus is still not satisfactory (Schlick-Steiner et al. 2006; Steiner et al. 2018; Salata and Borowiec 2019). However, a recent study (Steiner et al. 2018) suggested that genomic resources are essential for resolving the taxonomic problem. The harvesting ant, *Messor structor* (Latreille, 1798) is a keystone species (Arthofer et al. 2005). So far, only partial mitochondrial DNA sequences of this species have been reported (Arthofer et al. 2005; Steiner et al. 2018). A complete mitochondrial genome of this species can provide useful data on phylogenetic relationships of *Messor*. Here, we present the first complete mitogenome for *M*. *structor*.

The ant *M. structor* was collected from Gaochang county, Tulufan City, Xinjiang Province, China (N: 42°55′58.22″, E: 89°11′53.60″). The specimen and DNA sample of *M. structor* were deposited in the Herbarium of Southwest Forestry University (http://bbg.swfu.edu.cn/, contact person: Xin-Min Zhang, zhangxm7908@163.com) under the voucher number B19-328). Ethics statement: This study is not applicable because the ant is not the regulated invertebrate animal. The total genomic DNA from worker ants was extracted using a modified cetyltrimethyl ammonium bromide (CTAB) procedure (Doyle and Doyle 1987). Sequencing library was constructed using ILLUMINA TruSeq^TM^ Nano DNA Library Preparation Kit (Illumina, San Diego, USA) according to the manufacturer's recommendations. Sequencing was carried out on the Illumina NovaSeq 6000 platform (TSINGKE Co., Ltd, Beijing, China). The mitochondrial genome of *M. structor* was *de novo* assembled using GetOrganelle v1.6.4 and SPAdes version 3.13.1. The mitogenome was annotated using the MITOS Web Server (Bernt et al. 2013), and then submitted to GenBank (accession number OL581665).

The complete mitogenome of *M*. *structo*r was 17,628 bp in length. The nucleotide composition was AT-biased (84.07%). The mitogenome contained 13 protein-coding genes (PCGs), two ribosomal RNA genes (rRNAs), 22 transfer RNA genes (tRNAs). The PCGs used ATA, ATG, or ATT as the start condon, and TAA or TAG as the stop condon. The tRNAs, ranging in size from 54-76 bp with a total length of 1520 bp.

To detect its phylogenetic relationships, the sequences of *M*. *structo*r, other 29 ants and two outgroup species (*Apis mellifera ligustica* and *Vespa mandarinia*) (downloaded from NCBI GenBank) were aligned by MAFFT v7.450 software (Katoh and Standley 2013). The phylogenetic tree was constructed by IQ-tree (v2.1.3, http://www.iqtree.org/) with the maximum-likelihood (ML) method. The model GTR + G + I was selected for ML analyses with 5000 ultrafast bootstraps replicates. Results showed that *M*. *structo*r was closely related to *Aphaenogaster famelica* (Figure 1). The new complete mitochondrial genome of *M*. *structor* will be a milestone in understanding phylogenetic relationship of the subfamily Myrmicinae species.

**Figure 1.**
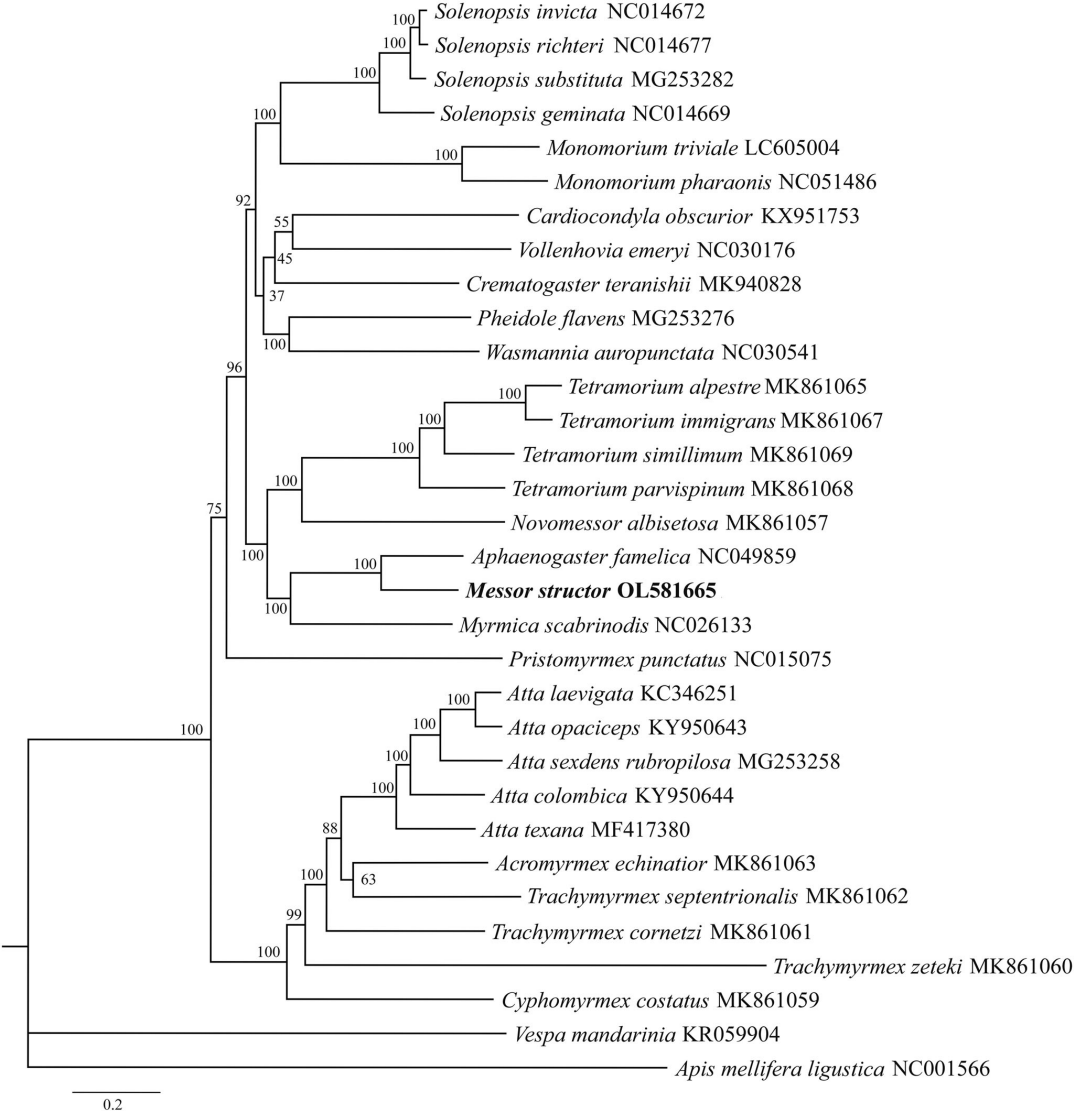
Phylogenetic position of *Messor structor* inferred by maximum likelihood (ML) based on the complete mitochondrial genome sequences. Bootstrap values are shown next to the nodes.

## Data Availability

The genome sequence data that support the findings of this study are openly available in GenBank of NCBI at https://www.ncbi.nlm.nih.gov under the accession no. OL581665. The associated BioProject, SRA, and Bio-Sample numbers are PRJNA785281, SAMN23553037 and SRR17085741, respectively.
